# Percutaneous Mechanical Circulatory Support in Acute Heart Failure Complicated with Cardiogenic Shock

**DOI:** 10.3390/jcm13092642

**Published:** 2024-04-30

**Authors:** Maria Parthena Xenitopoulou, Kyriaki Ziampa, Alexandros P. Evangeliou, Stergios Tzikas, Vasileios Vassilikos

**Affiliations:** 3rd Department of Cardiology, Aristotle University of Thessaloniki, 546 42 Thessaloniki, Greece

**Keywords:** percutaneous mechanical circulatory support, LVAD, cardiogenic shock, MCS devices

## Abstract

Despite advancements in algorithms concerning the management of cardiogenic shock, current guidelines still lack the adequate integration of mechanical circulatory support devices. In recent years, more and more devices have been developed to provide circulatory with or without respiratory support, when conservative treatment with inotropic agents and vasopressors has failed. Mechanical circulatory support can be contemplated for patients with severe, refractory, or acute-coronary-syndrome-related cardiogenic shock. Through this narrative review, we delve into the differences among the types of currently used devices by presenting their notable advantages and inconveniences. We address the technical issues emerging while choosing the best possible device, temporarily as a bridge to another treatment plan or as a destination therapy, in the optimal timing for each type of patient. We also highlight the diverse implantation and removal techniques to avoid major complications such as bleeding and limb ischemia. Ultimately, we hope to shed some light in the gaps of evidence and the importance of conducting further organized studies around the topic of mechanical circulatory support when dealing with such a high mortality rate.

## 1. Introduction

**Figure 1:**
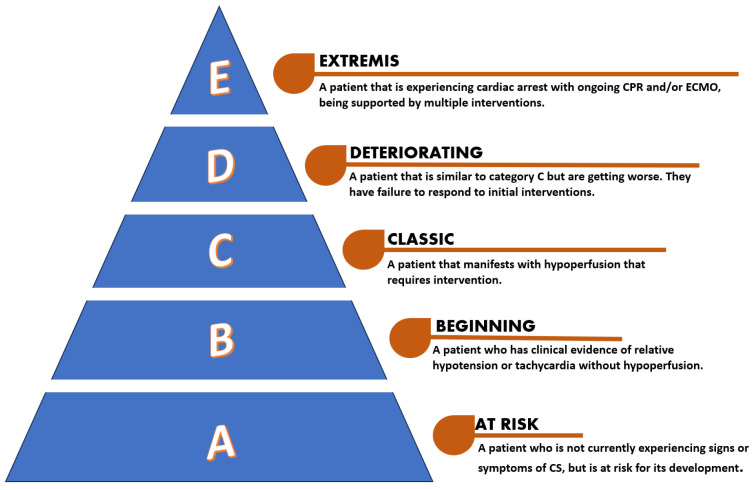
SCAI classification for cardiogenic shock [4].

Cardiogenic shock (CS) is a complex and critical clinical condition characterized by a significant reduction in cardiac output (CO), leading to an insufficient blood supply to organs and peripheral tissues, which can result in multiple organ dysfunction syndrome (MODS) [1]. Although acute myocardial infarction (AMI) is recognized as the primary cause of CS, the latter can also manifest in individuals with heart failure (HF) resulting from prolonged ventricular dysfunction such as acute decompensated heart failure with CS (ADHF-CS) [2]. CS is defined by a systolic blood pressure (SBP) of <90 mm Hg with adequate volume and clinical or laboratory signs of hypoperfusion [3]. Clinical hypoperfusion can be diagnosed by symptoms and signs such as cold extremities, oliguria, mental confusion, dizziness, and narrow pulse pressure, whereas laboratory findings that indicate hypoperfusion are, among others, metabolic acidosis, elevated serum lactate, and elevated serum creatinine. According to the Society for Cardiovascular Angiography and Interventions (SCAI) clinical expert consensus statement, CS can be classified from A to E depending on the severity of the patient’s clinical condition (Figure 1) [4]. 

Despite advancements in diagnostic and therapeutic approaches, CS remains a medical emergency with a high mortality rate exceeding 40% [2].

## 2. Current Management of CS 

Currently, the management of CS consists of circulatory support, pharmacological or mechanical, to ameliorate organ perfusion and increase the CO. Pharmacologic agents preferred for CS include an inotropic agent, such as dobutamine (Class IIb, Level of evidence: C) and a vasopressor such as norepinephrine (Class IIb, Level of evidence: B), often combining the two categories. The data comparing various supportive therapies exhibit inconsistencies, with none proving significantly beneficial in terms of major outcomes. Levosimendan seems to be the sole agent demonstrating a potential decrease in mortality compared to a placebo during the early stages of CS, even though it is not included in the current guidelines with a level of evidence [5,6]. However, it does not show a benefit when compared to other inotropes. Existing evidence regarding vasopressors indicates that norepinephrine may be linked to lower mortality rates, compared with dopamine or epinephrine [5,7,8]. PDE3 inhibitors can also be used in patients characterized by the absence of ischemia [3]. Notwithstanding, pharmacologic treatments should be customized for each individual and directed based on invasive hemodynamic monitoring.

In patients with CS due to ST-Elevation MI (STEMI), both ESC and the American Heart Association (AHA) propose early coronary angiography with the intention of proceeding to coronary revascularization (Class I, Level of evidence: C) [1,3]. 

Mechanical circulatory support (MCS) might be contemplated for specific patients with acute coronary syndrome (ACS) and severe or refractory CS. The use of left ventricular assist devices (LVADs) in those patients (Class IIb, Level of evidence C), depends on their age, comorbidities, and neurological function [3,9,10]. Unfortunately, there is limited evidence available to provide guidance on the optimal timing or selection criteria among this population. The first crucial step appears to be the accurate diagnosis of the CS SCAI stage using therapies recommended by guidelines and identifying patients likely to deteriorate rapidly for prompt transfer. Additionally, it is beneficial to recognize three distinct CS phenotypes upon initial presentation, non-congested, cardiorenal, and cardiometabolic, among patients with AMI-CS and Acute HF-CS.

## 3. Mechanical Circulatory Support Devices

MCS devices can be broadly categorized into temporary and durable types. Temporary MCS devices are either percutaneously or surgically inserted and serve as a bridge to recovery (BTR), where the device is removed after improvement in cardiac function; a bridge to a bridge (BTB), where a temporary device is used with a plan to transition to durable MCS after clinical stabilization; a bridge to transplantation (BTT) or a bridge to candidacy (BTC) for transplantation; or a bridge to decision (BTD). In the latter scenario, hemodynamic instability or medical complications of cardiogenic shock, such as neurological uncertainty or multisystem organ failure, may impede a comprehensive assessment for a durable MCS device or transplantation. The insertion of a temporary MCS device as a BTD allows for hemodynamic optimization, a potential reversal of cardiogenic shock-induced organ failure, and additional time for a thorough medical and social assessment before deciding on definitive therapies or opting for palliative care. On the other hand, durable MCS devices, which are surgically implanted, can be utilized as a BTR, as a BTT, or as a destination therapy (DT), in patients ineligible for transplantation or in long-term patients awaiting a heart transplant [1,3]. Table 1 summarizes the different MCS devices and their typical uses [3].

In terms of mechanical circulatory support, the IABP-SHOCK II trial proved that the application of intra-aortic balloon counterpulsation did not lead to a significant decrease in 30-day mortality among patients with CS as a complication of AMI, but mortality rates at 6 and 12 months were significantly lower in patients subdued to emergency revascularization [9]. Therefore, current ESC guidelines propose the non-qualification of the intra-aortic balloon pump (IABP) for patients with cardiogenic shock without mechanical complications as a routine practice (Class III, Level of evidence: B) [3]. 

While the new trend follows the percutaneous insertion of MCS devices, their application through a median sternotomy is typically recommended when peripheral systems fail to provide sufficient hemodynamic support or in patients with post-cardiotomy CS [11].

## 4. Extracorporeal Membrane Oxygenation (ECMO)

ECMO constitutes the initial device capable of providing both circulatory and respiratory support. The first successful use of ECMO was reported by Dr. Hill in 1972 [3]. A 24-year-old male patient with a sub-adventitial transection of the thoracic aorta from blunt trauma developed respiratory failure four days after the successful repair of the thoracic aorta [12]. In contemporary ECMO systems, essential components include the oxygenator for gas exchange, inflow and outflow tubing, the pump, and a hand crank. The closed ECMO circuit percutaneously withdraws deoxygenated blood from the right atrium or a central vein through the femoral vein and returns oxygenated blood through a second cannula to the arterial or venous system. The venous–arterial (V-A) ECMO system reintroduces fully saturated and decarboxylated blood into the descending aorta via the femoral artery. Cannulating the femoral artery is a challenging process that requires precise and careful manipulation to avoid limb ischemia [13]. Another factor to consider is the watershed phenomenon, where antegrade blood flow from the heart competes with retrograde oxygenated blood flow from the ECMO cannula in the descending aorta. When the watershed phenomenon occurs low in the thoracic aorta, the retrograde oxygenated blood from the ECMO does not contribute to coronary and cerebral blood perfusion.

The use of ECMO as a transition to advanced therapies has risen over the past ten years, driven by technological advancements, greater accessibility, increased familiarity among medical staff, the capacity to offer biventricular support, and the convenience of implantation in the catheterization lab [14]. The primary indication for ECMO use remains cardiopulmonary failure following cardiothoracic surgery, known as post-cardiotomy CS [15].

While the efficacy of ECMO implementation in AMI-induced CS remains a subject of debate [16], its proven value lies in serving as a temporary solution for refractory CS [17]. The ECMO-CS trial sought to evaluate the direct application of V-A ECMO versus an initially conservative approach in a total of 122 patients experiencing rapidly worsening or severe cardiogenic shock. It was concluded that the immediate implementation of V-A ECMO in this population did not improve clinical outcomes compared with an early conservative strategy that permitted the subsequent use of VA-ECMO in the case of worsening hemodynamic status [18].

Furthermore, findings from the ECLS-SHOCK trial indicate that the regular application of V-A ECMO in cases of AMI-CS fails to enhance 30-day survival and subjects patients to the potential hazards of bleeding complications and leg ischemia. This trial included participants with both STEMI and NSTEMI. Even among the high-risk population included in the study (median lactate 6.8 mmol/L), no discernible subgroups demonstrated any benefit from the use of V-A ECMO [19,20].

An extensive contemporary study on V-A ECMO in cardiogenic shock revealed a substantial 23-fold surge in its utilization from 2002 to 2018, with 25,621 (1.5%) of 1,633,877 patients with CS receiving circulatory support with ECMO. A notable 35% reduction in mortality over the study period was observed, although patients receiving V-A ECMO were characterized by a younger age and fewer co-morbidities. It is evident that this could also be attributed to numerous other factors such as the increased familiarity of ECMO use among the medical stuff, the modernization of the device itself, etc. Through this research, it was revealed that, among the 25,621 patients who received VA-ECMO, 56% died, 6% were bridged to a permanent LVAD, and 2.5% were bridged to transplant, while approximately 36% recovered without a need for LVAD or transplant. 

Additionally, through this registry, it was revealed that only a minority of patients on V-A ECMO are transitioned to either LVAD or heart transplant, and approximately one-third of patients recover without necessitating heart replacement therapy such as LVAD or transplant [21].

The utilization of ECMO as a transitional support to advanced therapies has risen over time, with a greater number of patients being guided for LVAD over orthotopic heart transplant (OHT). Data from the Extracorporeal Life Support Organization of patients with cardiogenic shock that were bridged with ECMO to OHT or LVAD from 2010 to 2019 revealed that mortality was equal between the LVAD group and the OHT one, despite the worse clinical presentation of the first group of patients. Despite equal mortality rates between the two groups, patients undergoing OHT experienced a longer length of stay [14].

ECMO is also applied as a bridge to lung transplantation in cases of lung disease [22] and as a bridge to heart transplantation in fulminant myocarditis [23]. Another variation, Veno-Venous ECMO, is employed in conditions resembling severe respiratory failure [24,25]. 

## 5. Impella

The Impella series comprises percutaneously catheter-based mechanical circulatory assist devices designed to provide up to 6.0L of cardiac output and reduce LV preload. These devices are inserted through central arteries, such as the femoral or axillary artery, and are placed across the aortic valve into the LV. It then facilitates blood flow from the left ventricle into the ascending aorta. The first transvalvular microaxial flow pump was called the Hemopump (Nimbus) and was first implanted in 1988 [26]. The newest trans-valvular micro-axial flow pump models reaching these targets are the Impella 5.0 and 5.5 [27]. There are various types of Impella devices with differing stroke volumes (SVs), as outlined in Table 1. 

The Impella RP is recommended for cases of right ventricular (RV) failure following LVAD implantation, AMI, or acute pulmonary embolism (PE) [28,29,30]. In individuals with end-stage HF or severe myocarditis, Impella appears to be highly effective as a BTR, a BTT, or a bridge to a surgical heart pump implantation [31,32]. Moreover, Impella shows potential in aiding the stabilization of patients with aortic valve stenosis in need of transcatheter aortic valve implantation [33]. Several published studies have demonstrated Impella’s noteworthy circulatory support and its ability to provide anti-ischemic myocardial protection in high-risk percutaneous coronary intervention (PCI) settings [34,35,36,37]. Recent research data suggest the potential use of Impella devices in patients with CS or cardiac arrest resulting from AMI to enhance outcomes [38,39]. Early Impella implantation prior to PCI is associated with higher survival rates and those of successful revascularization [40]. However, the role of Impella in AMI-induced CS remains a contentious issue, as indicated in a recently published meta-analysis that included 17 studies and 3933 patients [41]. It was revealed that 30-day mortality (primary endpoint) in patients with CS treated with Impella was 47.8%; nevertheless, the use of Impella CP or 5.0, their positioning before revascularization, or their application in patients without cardiac arrest (CA) is linked to decreased mortality. Another recent single-center study suggests elevated mortality rates despite the use of Impella. A total of 172 patients were included and received Impella therapy for cardiogenic shock with the 30-day mortality being 56.2% and the 6-month mortality being 60.7% [42].

A 2024 international, multicenter, randomized trial of 355 patients with STEMI complicated with CS compared the use of Impella CP before, during, or up to 12 h after primary PCI versus the standard of care. Interestingly, it was reported that the use of Impella led to a lower risk of death from any cause within 180 days, which was the primary endpoint. The number that needed to be treated to avoid one death was eight. There was also a reduction in the composite secondary endpoint of additional mechanical heart support, heart transplant, or death with the heart pump. However, these favorable results were in cost of a higher completion rate in the Impella group [43]. 

## 6. iVAC2L

The PulseCath iVAC2L (PulseCath BV, Amsterdam, the Netherlands) is an innovative pulsatile circulatory support device designed to receive blood from the LV and eject it in the ascending aorta. Among its components is a flow catheter with an extracorporeal pneumatic dual chamber and a patented rotating two-way valve. The device is equipped with an extracorporeal membrane pump linked to a mainstream IABP console. This console serves as a pneumatic driver for the pump, enabling it to generate an output of up to 2.0 L/min. The pulsatile action of the iVAC2L operates in synchrony with the cardiac cycle, facilitating blood aspiration from the LV during cardiac systole. However, at higher heart rates, the output may be reduced as the foreshortened diastolic phase does not provide sufficient time for effective ejection [44]. The primary indication for the use of the iVAC2L device is CS complicating an AMI. Additionally, its application has extended to include patients with CS post-cardiac surgery or induced by viral infections. The device has also found utility in preventing hemodynamic instability during high-risk PCI procedures [45,46,47]. 

The device has been designed to temporarily support the LV after percutaneous insertion through the femoral artery, and, oftentimes, for RV support, with the insertion through the pulmonary artery trunk, a procedure typically performed during cardiac surgery [48]. A comprehensive review of the available literature revealed numerous studies and case reports [46,47,48,49,50,51,52,53,54], highlighting the use of the iVAC2L device for circulatory support, with indications of use, type of vascular access, and pre- and post-implantation hemodynamical parameters, as well as potential complications of the implantation.

A recent trial, which involved 20 patients undergoing circulatory support via the iVAC2L during high-risk PCI, assessed aortic pressure data following device insertion and immediately post-intervention. The findings suggest that the procedure is both feasible and safe, with indications that aortic pressure rises with continued support [55].

## 7. Combination Therapy

In challenging scenarios such as patients on ECMO experiencing shock, pulmonary edema, and left ventricular failure requiring left ventricular decompression, a combination of ECMO and iVAC2L or Impella may offer benefits. This approach aims to minimize the impact of the watershed phenomenon, preventing coronary and cerebral hypoperfusion while enhancing cardiac output [49,50].

This scenario was studied in a recent meta-analysis comparing the use of V-A ΕCMO with or without Impella in patients with CS. It was revealed that the simultaneous unloading of the LV using Impella was associated with a reduced short-term mortality and an increased likelihood of transitioning to a durable LVAD or heart transplant [56]. However, patients supported with ECMELLA (ECMO and Impella) faced elevated incidences of hemolysis, limb ischemia, and renal failure necessitating continuous renal replacement therapy. 

A large meta-analysis comparing the use of ECMO versus Impella indicated that, among patients with CS, the utilization of Impella was linked to reduced in-hospital mortality, stroke, and device-related complications compared to ECMO. However, these results could be questioned given the fact that patients treated with Impella had lower baseline lactate levels in comparison to those treated with ECMO [57]. Prospective, well-organized, multicenter trials currently in progress are anticipated to provide clarification and address the existing data gaps [58].

## 8. Limitations/Contraindications

Admittedly, the use of MCS devices comes with a number of limitations. Impella’s disadvantage lies in the lack of respiratory support, mandatory anticoagulation, and the increased risk of hemolysis and peripheral ischemia, as well as the increased cost. Contraindications for the use of Impella include the presence of RV failure, LV thrombus, a prosthetic aortic valve, moderate aortic stenosis/aortic regurgitation, severe aortic disease, and a contraindication to anticoagulation and that of a ventricular septal defect. Extracorporeal life support such as ECMO presents its own limitations such as the need for perfusionist support, LV distention, increased peripheral ischemia, venous thrombosis, and the risk of cerebral hypoxia. ECLS is generally contraindicated in severe aortic regurgitation, severe aortic disease, and in cases where there is a contraindication to anticoagulation [11].

## 9. Patient Selection

The patient selection criteria are shaped by the interplay of effectiveness, institutional proficiency, and device-associated complications. Choosing patients for percutaneous MCS in CS requires th ecareful consideration of several factors: (a) the etiology and the severity of CS, (b) the unresponsiveness to conventional therapy, (c) anatomical and hemodynamic considerations involving the feasibility of vascular access, (d) comorbidities and the overall prognosis of the patients, (e) expertise and institutional resources involving the presence of a multidisciplinary team experienced in MCS management, and, finally, (f) cost. Ultimately, the decision to initiate MCS in cardiogenic shock requires careful clinical judgment, multidisciplinary collaboration, and a consideration of individual patient characteristics and preferences. It is essential to continuously reassess the patient’s response to therapy and adjust management accordingly. 

The INTERMACS classification is a valuable tool in patient selection according to their profile. The database is currently being used to assess the survival and complication rates of different patient profiles receiving MCS therapy. Data from the “The Society of Thoracic Surgeons” Intermacs database annual report suggests that the patient profile has shifted to include a higher percentage of individuals classified as profile 3, “inotrope-dependent” patients with stable blood pressure and end-organ function (26% from 2006 to 2011 compared to 35% from 2012 to 2016), with a decrease in those classified as profile 2, patients in “progressive decline” with worsening end-organ function on intravenous inotropes (40% versus 35%). Additionally, there has been an increase in patients with better markers of preoperative renal and hepatic function and more patients receiving implants for destination therapy (29% versus 48%) [59].

## 10. Implantation Timing 

Undoubtedly, in any case, a patient in CS requires early recognition, resuscitation, and stabilization; attentive monitoring is of critical importance, whilst fluid challenge and adequate support with vasopressors and inotropic agents are considered as first-line therapeutic options. MCS may also be necessary to ensure systemic perfusion, stabilize the hemodynamic status, and serve as a bridge to further therapeutic interventions [3,60,61]. However, the optimal timing for implementing MCS in CS patients remains a matter of controversy [62]. 

A recent large analysis of the National Inpatient Sample database of the United States revealed that, in non-AMI-related CS, and based on survival to 24 h after admission, the early initiation of MCS had a statistically significant decrease in all-cause hospital mortality, along with a lower incidence of vascular and renal complications, and a shorter hospital stay. Concurrently, the delayed initiation of MCS correlated with a higher incidence of advanced therapeutic interventions, such as LVAD implantation and transplantation [63].

The development and validation of an algorithm capable of predicting the progression of CS and assisting physicians in the optimal placement of an MCS device are of the utmost importance. Providing hemodynamic support in the early stages of CS has been proven to offer significant benefits for the patient with the most important one being a substantial extension of time aiming at a potential recovery [1].

## 11. Complications of Use and Removal of the Device

A major challenge with percutaneous MCS devices is the access site for vascular catheter insertion to avoid complications during or after sheath removal. These complications depend on factors such as the device size, vessel diameter, and patient’s medical history. It is crucial that the removal of the cannula is performed by a trained physician, as it may lead to major complications. These include bleeding and artery perforation, requiring surgical reconstruction. 

Novel vascular closure devices (VCDs) for large-bore femoral arterial punctures have been developed, offering a valuable means of ensuring safe hemostasis. The deployment of VCDs is recommended prior to introducing a large-bore access sheath. This system operates by using needles to apply, attach, and withdraw sutures through the same path in the arterial wall. Once the suture is securely deployed, the arteriotomy can be closed, and the knot can be secured. A meta-analysis revealed that the use of these closure devices was associated with a significantly lower incidence of groin hematomas or pseudoaneurysms, as well as a shorter hemostasis time compared to extrinsic compression [64]. A recent retrospective cohort study examined the complications percentage after the placement of Perclose ProGlide (Abbott Vascular, Temecula, CA, USA), a percutaneous suture-mediated closure system, and MANTA VCD (Teleflex Vascular, Wayne, PA, USA), a collagen-based closure device. It was shown that, in the group of patients to whom Perclose ProGlide was used, there was a significantly less severe complication rate. In contrast, the MANTA VCD group exhibited a higher frequency of major bleeding complications, necessitating a more complex treatment with a potentially more significant impact on quality of life [65]. Another viable solution always remains the surgical removal of the device. 

In cases of AMI-CS, there is a heightened risk of bleeding, which is correlated with unfavorable outcomes. The utilization of an LVAD is identified as one of the factors predicting bleeding events in such patients. Bleeding incidents can be categorized into access-site and non-access-site bleeding. The latter is associated with factors such as thrombocytopenia, sepsis, shear–stress-induced acquired von Willebrand syndrome, and the administration of anticoagulation therapies [5]. The axial flow Impella devices, unlike the pulsatile iVAC, come with the drawback of a substantially higher risk of hemolysis (seen in 10% of patients within the first 24 h) due to the rapid rotation of the axial flow pump [66,67,68,69,70]. Hemolysis has been associated with a poorer prognosis among ACS patients [71,72,73]. Tschope et al. documented a case of significant hemolysis following the ECMELLA approach. Substituting Impella with iVAC effectively supported the patient’s hemodynamic status and resulted in a milder hemolytic effect [50]. Albeit the increased bleeding risk, a recent meta-analysis of the ECMELLA versus ECMO-only strategies for the management of CS suggests that the first may reduce 30-day mortality and increase LV recovery [41].

Limb ischemia is another common problem in prolonged large-bore access. Specifically, in VA-ECMO or ECMELLA, it becomes imperative to use antegrade perfusion techniques to ensure sufficient blood flow to the distal limb. When encountering limb ischemia with the exclusive use of mAFP, it may be advisable to investigate vasospasm as a potential cause of ischemia [5].

## 12. Cost-Effectiveness

As indicated in Table 1, the utilization of LVADs may be constrained by their higher cost relative to other MCS devices. According to research led by Stretch et al., PVADs were shown to decrease expenses by $45,000 and $54,000 per case in instances of cardiogenic shock stemming from myocardial infarction and other heart conditions, respectively [74]. Additionally, they were associated with a 58% decrease in mortality rates.

In cases of cardiogenic shock necessitating immediate hemodynamic assistance, PVAD therapy led to improved results, reduced hospital stays, and decreased expenses, and conferred a survival advantage compared to surgical hemodynamic support alternatives [75]. 

On the other hand, ECMO proved not to be cost-effective in a study comparing the incremental cost-effectiveness ratio between those who received ECMO as a bridge to LVAD or BTT and those who did not [76].

Overall short-term mechanical circulatory support seems to be cost-effective, while long-term mechanical circulatory support as a bridge to transplantation or destination therapy lacks supporting data on being cost-effective. 

## 13. Discussion 

Over the past few decades, advancements in technology have transformed mechanical MCS devices into pivotal tools for managing CS and providing hemodynamic support during high-risk PCI procedures. These devices not only help prevent circulatory collapse but also offer valuable time for patients to potentially recover from reversible conditions. As previously discussed, percutaneously inserted LVADs have been utilized not only for LV support but also for RV support in patients experiencing post-cardiac surgery CS [48]. 

Recent advancements include the simultaneous use of LVADs with V-A ECMO as evidenced by a newly published case that supports the concept of potential future applications of dual-assist devices. Approaches such as EC-iVAC (simultaneous use of ECMO and iVAC) and ECMELLA (simultaneous use of ECMO and Impella) may be considered as options for patients in CS requiring immediate LV support [49,50]. The ECMELLA approach has recently been a topic of interest. This strategy is implemented for patients with CS who show worsening despite receiving treatment with a mAFP. In such cases, additional perfusion is introduced through V-A ECMO. Alternatively, this approach is adopted as an initial treatment strategy for patients facing severe cardiogenic shock and are at risk of left ventricular distension or overload. As patients clinically stabilize and myocardial recovery is observed, these devices can be gradually or simultaneously reduced and eventually discontinued [19]. Another common scenario implementing the ECMELLA strategy concerns patients in SCAI shock stages D and E facing imminent cardiac and circulatory collapse that necessitate the urgent implementation of V-A ECMO to ensure tissue perfusion and oxygenation. Simultaneously, the use of MCS devices like mAFP serves the dual purpose of unloading the LV and aiding in myocardial recovery. Recent data also suggest that the mortality risk appears to be even lower when the mAFP is implanted at an earlier stage, ideally before or concurrently with V-A ECMO placement [19]. 

The concept of protected weaning has been suggested, taking into account clinical indications, hemodynamic stability, laboratory measurements, and imaging [19]. The de-escalation strategy defined as the strategy aiming at the prompt discontinuation of V-A ECMO to reduce the escalating complications associated with prolonged ECMO use has also been proposed. De-escalation becomes a viable strategy once the shock parameters have shown improvement, inotropic support has been successfully tapered, and the patient is euvolemic. The timing for executing de-escalation is aligned with the recovery of shock parameters and the achievement of hemodynamic stability [77].

The complex case of mixed CS (both cardiogenic and septic) poses an evident challenge. While temporary MCS is considered a viable choice for managing cardiogenic shock associated with sepsis, the key lies in identifying patients whose shock predominantly stems from cardiac issues rather than sepsis itself. The successful implementation of this approach, particularly in the absence of clinical trials, hinges on accurately selecting such patients. The key is to implement such a strategy in patients with pre-existing HF who enter septic shock that cannot be compensated by the limited reserves. Another possible case of a mixed shock where MCS could have a place is for patients with AMI-related CS complicated with acute infection after cardiac arrest per se. The increased risk of infection with the use of MCS devices is also noteworthy in a setting of septic shock, while careful consideration is vital due to potential interactions with medications used for sepsis [78]. However, case reports such as the use of iVAC2L implantation in a patient with a mixed cardiogenic and septic shock with Influenza type A and the combination of ECMO with cytokine removal therapy in a patient with cardiogenic septic shock could lead the way for organized trials aiming at the broadened use of MCS [47,79].

## 14. Conclusions

In summary, there is a lack of clinical studies directly comparing MCS devices, and their simultaneous or consequent use while applying different strategies among diverse patient groups. Further research is needed to address these gaps in evidence. The multi-device approaches that are widely utilized throughout various phases of CS in the present era are actively being examined in ongoing RCTs [80]. It is crucial that a complete heart team is responsible for the management of challenging cases like this. Ultimately, there is hope that the results of these studies will aid in the development of consistent algorithms that will help navigate the complex cases of CS, offering guidance on device selection and implantation methods based on the hemodynamic status and individual history of each patient.

## Figures and Tables

**Table 1:** Different percutaneous mechanical circulatory support devices and their characteristics.

	V-A ECMO	Impella^®^ 2.5 L	Impella^®^ 5.5 L	Impella^®^ CP	iVAC^®^ 2.0 L
Catheter size (F)	-	9	9	9	11
Cannula size (F)	14–19 arterial 17–21 venous	12	19	14	17
Maximum flow (L/min)	7.0	2.5	6.0	4.3	2.0
Pump mechanism	Centrifugal flow—continuous pump	Axial flow—continuous pump	Axial flow—continuous pump	Axial flow—continuous pump	Pulsatile flow—depends on HR
Blood is aspirated from	Right Atrium	Left Ventricle	Left Ventricle	Left Ventricle	Left Ventricle
Blood is ejected in	Aorta	Ascending Aorta	Ascending Aorta	Ascending Aorta	Aorta
Access site	Femoral Vein Femoral Artery	Femoral Artery	Axillary Artery	Femoral Artery	Femoral Artery
Implantation	Percutaneous	Percutaneous	Percutaneous	Percutaneous	Percutaneous
LV support		+	+	+	+
Indications		Advanced HF Cardiogenic shock Postcardiotomy cardiogenic shock High-risk PCI	Cardiogenic shock Postcardiotomy cardiogenic shock	Advanced HF Cardiogenic shock Postcardiotomy cardiogenic shock High-risk PCI	High-risk PCI Acute myocardial infarction Cardiogenic shock Left ventricular failure with an EF < 30%, and/or CI < 2.5 L/min/m^2^
Maximal duration of application		Up to 4 days	Up to 14 days	Up to 4 days	Up to 24 h
Cost	+ (+)	+++	++++	++++	++

## Data Availability

No new data were created or analyzed in this study. Data sharing is not applicable to this article.
